# Effect of a Combined Program of Strength and Dual Cognitive-Motor Tasks in Multiple Sclerosis Subjects

**DOI:** 10.3390/ijerph17176397

**Published:** 2020-09-02

**Authors:** Carmen Gutiérrez-Cruz, F. Javier Rojas-Ruiz, Juan Carlos De la Cruz-Márquez, Marcos Gutiérrez-Dávila

**Affiliations:** Department of Physical Education and Sport, University of Granada, 18071 Granada, Spain; carmengc90@hotmail.com (C.G.-C.); dlcruz@ugr.es (J.C.D.l.C.-M.); marcosgd@ugr.es (M.G.-D.)

**Keywords:** biomechanics, force, balance, gait, dual tasking

## Abstract

This study investigated the effects of a 24-week combined training program (CTP) based on strength exercises and cognitive–motor tasks performed concurrently in participants with multiple sclerosis. A randomized, controlled intervention study was carried out. In total, 31 subjects with a confirmed diagnosis of multiple sclerosis (14 men and 17 women) were stratified and randomized into an intervention group (17 subjects) and a control group (14 subjects). The intervention group completed three weekly training sessions for 24 weeks, while the control group pursued their normal daily activities. In this program, cognitive–motor tasks were completed at once (dual tasking). A 3D photogrammetry connected to a selective attention system designed for dual tasking while walking was used. Ground reaction forces were measured using two force plates, one for sit-to-stand testing, while the other was used for static force measurement. Postural equilibrium was examined using a stabilometric plate based for Romberg test assessment. The 24-week training program for multiple sclerosis patients improved their static peak force by 11% (*p* < 0 .05), their rate of force development by 36% (*p* < 0.05), and their balance (*p* < 0.05). Performance in daily activities such as walking or sitting-to-standing improved significantly in multiple sclerosis participants. CTP training was effective in reducing the dual-task costs of step length (48%) and walking velocity (54%), as compared to a matched control group.

## 1. Introduction

Multiple sclerosis (MS) is a chronic inflammatory neurodegenerative disease that causes the demyelization of the nervous fibers of the encephalus and the spinal cord. This neurological process causes unpredictable alterations in the motor, sensory and cognitive systems, and associated physical and mental comorbidities [1,2,3]. Although the effectiveness of rehabilitation and pharmacological interventions for MS has improved substantially in the recent decades, these interventions are only aimed at delaying the progress of the disease [4]. Physical exercise has been consistently demonstrated to have therapeutic effects in MS subjects, as it improves MS-associated motor and cognitive symptoms, including muscle weakness [5,6], balance and coordination [7,8], muscle fatigue [9,10], vascular and metabolic comorbidities [11,12], cognitive/motor dysfunction [13,14], and more recently the pathophysiology of the disease [15,16]. 

Therefore, the evidence obtained to date suggests that physical exercise improves the quality of life of MS subjects. Yet, no standard physical exercise programs have been developed, but a range of physical exercise programs have been proposed [17]. These programs included vibratory stimulation training, aquatic exercises or yoga [18,19,20]. However, most of these training programs are aimed at improving muscle mechanics (force, power and resistance) to recover strength and postural control, which are the most common motor symptoms of MS. 

These programs are useful to treat motor dysfunction, but integral training interventions should also address cognitive deterioration. Neurological deterioration causes loss of muscle strength and postural control [21]. Dual tasking (DT) is generally used to assess the effects of cognitive impairment on motor performance. DT helps assess changes in motor activity patterns while a cognitive task is performed concurrently. Simultaneous activities compete for resources, which is defined as “dual-task cost” (DTC) [14,21]. 

The aim of this study was to examine the effects of a combined training program (CTP) based on strength exercises and cognitive–motor tasks performed concurrently by participants with multiple sclerosis. The effects were assessed on the kinematic factors of gait cycle, the cost of concurrent cognitive tasks, balance and static/dynamic peak force during sit-to-stand tests in MS subjects. All this exposed, it was hypothesized that all parameters described would improve after a 24-week training intervention program.

## 2. Materials and Methods 

### 2.1. Design 

A randomized, controlled intervention study was carried out. Subjects in the intervention group attended three CTP sessions for 24 weeks. Parameters were measured at baseline, prior to the intervention (Pre), at 12 weeks (Post) and at 24 weeks, when the intervention program finished (Repost). The control group carried out their normal daily activities, which included prescribed rehabilitation therapies. Assessments were only done in controls at baseline (Pre) and at 24 weeks (Repost). 

Most research studies were based on 8–12-week training programs [22]. This study was increased to 24 weeks, as a longer duration program will presumably have more significant effects on daily activity performance in MS subjects. This 24-week duration is supported by other authors, such as Fimland et al. [23] and Hosseini et al. [6].

### 2.2. Subjects 

The sample was composed of 31 subjects diagnosed with multiple sclerosis (14 men and 17 women). The inclusion criteria were as follows: (a) a confirmed diagnosis of MS with an Expanded Disability Status Scale (EDSS) score determined by Kurtzke of 0 to 6; (b) ability to walk without aid or assistance; (c) no history of surgery or fracture in the lower limbs within the last year; (d) absence of MS flare-ups within the last six months and (e) not suffering from chronic psychological or emotional conditions that affect motor coordination. All subjects completed the World Health Organization Quality of Life (WHOQOL-BREF) questionnaire for the evaluation of the quality of life of the participants, and underwent an analysis of body composition via the InBody-230 system that uses the impedance or resistance that the body offers to the passage of two currents with different frequencies (20 kHz and 100 kHz). Based on general data and previous assessments, the subjects were stratified and randomized either to the intervention group (8 men and 9 women—Total: 17 subjects) or to the control group (6 men and 9 women—Total: 14 subjects). Patients who underwent flare-ups, severe illness or surgery during the intervention period were excluded. Finally, the sample was composed of 15 subjects in the intervention group (7 men and 8 women) and 11 in the control group (5 men and 6 women). Table 1 shows the most relevant characteristics of subjects, wherein it is found that there were no statistically significant differences between the means of the two groups for any of the characteristics. The University of Granada conducted this study between September 2017 and June 2018. The study was granted ethical approval by the Human Research Ethics Committee of the University of Granada; the ethics approval number was 164/CEIH/2016 and the experimental procedures were also registered.

### 2.3. Combined Training Program (CTP)

Each subject attended three 60 min sessions weekly led by a coach who was specifically trained for this study. If a subject missed a session, the missed session was rescheduled during the same week. Most sessions were held in groups of 5–6 subjects grouped by level of disability. Sessions started with a general 5 min warm-up that included walking and joint mobility exercises. Sessions were completed with 5 min stretching. All exercises were adapted to the level of disability and individual characteristics of each subject.

The combined training program (CTP) was structured in four 10 min sets of exercises aimed at training different abilities: 

(a) General dynamic strength. This included static and dynamics strength exercises using the body as the only load. Progression was regulated by increasing the number of repetitions of each exercise; 

(b) Dynamic strength against resistance. The same as in general dynamic strength, but using bodybuilding machines, elastic bands or manual resistance. Progression was achieved by increasing the load and maintaining the same number of repetitions; 

(c) Dual walking and/or run. A walkway was connected to a computer-controlled electronic system composed of 6 lights (simulating two traffic lights) and a push button placed in front of the subject. Subjects were asked to walk or run while a sequence of lights was presented. When the red lights of the two “traffic lights” turned on simultaneously, the subject had to push the button as fast as possible. The system measured and recorded subject’s reaction time. Progression was modulated by increasing the walkway speed and maintaining the duration of the exercise; 

(d) Dual task on instable plates. Using a light system similar to that described above, subjects were asked to perform different coordination tasks while walking or running on instable plates. Exercise intensity was regulated by changing the level of difficulty in maintaining balance.

### 2.4. Materials and Measurement Systems 

Measurements were made on a 4.6 m walkway marked with a spatial reference system (RS) which consisted of 12 equidistant points placed in the center of the walkway (3.16 m long × 1.58 m wide × 1.68 m high) associated with other marks on the ground. Gait analysis was completed by 3D photogrammetry based on data provided by two high-speed cameras (JVC GC-PX100 BE) set at 200 Hz placed on the side of the walkway. The cameras were synchronized using an electronic signal that activated an LED placed within the active field of the two cameras. To assess gait under the dual-task condition, we forced the use of selective attention using a system simulating two traffic lights installed on a tripod at the end of the walkway at a 2 m distance from the subject, the geometric center of which was 1.70 m high. Each traffic light was composed of three LED lights 0.10 m in diameter. From top to bottom, the lights were red, yellow and green. The two traffic lights were connected to a computer with a programmable external card that controlled the activation of the six lights. 

The equipment used included four Dinascan/IBV 0.6 × 0.37 m force plates (Instituto de Biomecánica de Valencia, Valencia, Spain) set at 500 Hz. The surface of one of the plates was extended by attaching a wooden platform (0.6 × 0.9 m) on which a height-adjustable stool was installed [24]. The other force plate was placed in front of a force tower fixed to the floor which had a height-adjustable horizontal bar. 

Stabilometric parameters were measured using a force plate that recorded the pressure center, *PC* Dinascan/IBV, in Hz, connected to the balance assessment system DedSVE/IBV. For experimental conditions based on an instable plate, we used foam (Balance-pad Elite® foam HerexC70.40®, ISO 9001).

### 2.5. Interventions

Subjects were asked to answer some preliminary questionnaires. Then, each subject completed four sets of measurements (static strength, gait, balance and sit-to-stand task). Measurements were made in a random order.

#### 2.5.1. Static Strength 

After active warm-up, subjects stepped onto the force plate with the horizontal bar of the force tower positioned in front of them at a height of 120% of the distance of the knees from the ground (middle lateral epicondyle). Subjects were instructed to make a slight push-up with the trunk in an upright position, grip the bar, and stay in that position until the signal was delivered; when the LED light turned on, they had to rapidly try to pull up the bar, which was fixed, as hard as they could (countermovement was blocked). After several practice repetitions to adapt to the test, the subjects performed five valid trials. The vertical component of the ground reaction forces was measured; peak force was estimated as the median of the peak force values reached in the five trials (peak force _(Z)_). The rate of force development of the vertical component (RFD_(Z)_), as 30% of the peak force _(Z)_ divided by the time to peak force, was calculated.

#### 2.5.2. Gait Analysis

Each subject was instructed to walk in the following conditions: (a) Normal gait. In the normal gait condition, each subject was instructed to walk normally at a self-selected pace beginning at a distance of three meters from the electronic walkway; (b) Dual-task gait. The conditions were the same as in (a), but subjects were asked to stop walking as soon as the two red lights turned on. While walking, the lights randomly turned on and off. The experimental conditions were randomly presented to each subject. 

Data for each group and condition were collected from five valid trials. In the dual-task condition, the red lights never turned on simultaneously in the five valid trials. To force subjects to maintain selective attention, three further trials were completed where the two red lights turned on simultaneously. Measurements were not made during these three trials. Trials were performed in a random order. The analysis of records was based on data from the valid trial whereby gait cycle time (GCT) was the median of the five GCTs obtained in each experimental condition

Gait cycle was defined as the time interval between floor contact of the right heel and the following heel contact of the same limb [25]. The following parameters were measured: time to two consecutive ground contacts of the same foot (*GCT*); mean time between the two double-support and single-support phases of a gait cycle (double-support time and single-support time, respectively); mean horizontal distance between the two steps that compose a gait cycle, determined by three ground contacts of the heels of the two feet (step length); mean velocity of the CM during the gait cycle (VCG cycle); and mean velocity of the CM during double-support and single-support (VCG double-support and VCG single-support, respectively). 

The DTC for each group and variable was calculated from the percentage of variance between normal gait values (NG) and dual-task gait (DTG) [14].

$$DTC\%=\frac{\mathrm{NG}-\mathrm{DTG}}{\mathrm{NG}}\cdot 100$$

#### 2.5.3. Stabilometry

Stabilometry was based on the Romberg test. The subject stands on the force plate barefoot, with arms along the body and with their feet placed so as to maintain the heels together and a 30–35° angle between the right and left toes. Then, the subject is asked to stay as still as possible for 20 s. The test was performed under four experimental conditions: (a) with eyes open and on the rigid surface of the force plate (ROE); (b) with eyes closed (RCE); (c) on instable foam with eyes open (RFOE); and (d) on foam with eyes closed (RFCE). Three valid trials were performed in each experimental condition. We calculated the mean of the three values obtained for each parameter in each condition. Conditions were presented in a random order. In each condition, we measured the mean anterio-posterior and lateral displacement of the pressure center (*AP_(PC)_* and *ML_(PC)_*, respectively) and their total length (TOTAL *L_(PC))_*) 

#### 2.5.4. Sit-to-Stand (STS)

STS was performed according to the method described by Papa and Cappozzo (2000) [24]. After the force plate was calibrated to zero, subjects were instructed to sit on the seat, the height of which was adjusted to a height of 80% of the distance between the knees and the ground (head of the fibula), with their arms folded on their chest, the trunk in upright position and the feet parallel on the plate. When the signal was delivered, the subjects had to stand up rapidly without raising their shoulders or moving their feet, and stay motionless when they reached a vertical position. Five valid trials were executed. The vertical (F_Z_) and horizontal (F_X_) components of ground reaction forces were measured. We only considered for analysis the trial where STS time matched the median STS time of the five trials. Onset of movement was defined as the moment at which the base line of the vertical component (with subject in seating position) reached a value ±1% the weight of the subject. The end of the movement was defined as the moment at which vertical velocity of the CM was <0.05 ms ^−1^. To determine the onset of take-off from the seat, we synchronized a video camera with the force plate to measure the time interval between the onset of the movement and take-off (take-off time) and the time interval between take-off and completion of the movement (post-take-off time).

From the horizontal and vertical components of ground reaction forces, we calculated their respective peak forces (PF_(X)_ ad PF_(Z)_, respectively). The net vertical peak force (PF_N(Z)_) was calculated as the difference between PF_(Z)_ and the subject’s body weight. Vertical and horizontal acceleration components were estimated from their respective net force and the subject’s body mass. Records of the vertical and horizontal components of the velocity of the CM (_CM(Z)**V**_ and _CM(X)_**v**, respectively) were determined by the integral of acceleration–time functions using the trapezoidal method with a time increase of 0.005 s, and 0 was used as the constant of integration. 

### 2.6. Statistical Analysis

Means and standard deviation (SD) were calculated for each group and experimental situation. Differences between the means obtained in each period (Pre, Post and Repost for the intervention group, and Pre-Repost for controls) were calculated by a repeated-measures analysis of variance (ANOVA). Differences between groups were detected by multivariate analysis. A *p* < 0.05 was considered statistically significant. 

To assess gait and sit-to-stand test reliability, a repeated measures ANOVA for all trials (five valid trials for each experimental condition) was used, with gait cycle time and sit-to-stand time as the dependent variables, respectively. No significant differences were observed. In the pre-intervention assessment of the intervention group, the intraclass correlation coefficient (ICC) was 0.912 (*p <* 0.001) for normal gait and 0.897 (*p* < 0.001) for sit-to-stand. The intraclass correlation coefficients (ICCs) for the Control Group were 0.987 (*p* < 0.001) and 0.981 (*p* < 0.001), respectively. All statistical analyses were performed using the Statgraphic Plus 5.1 package for Windows (Statgraphics Technologies, Inc., The Plains, VA, USA). 

## 3. Results

**Figure 1:**
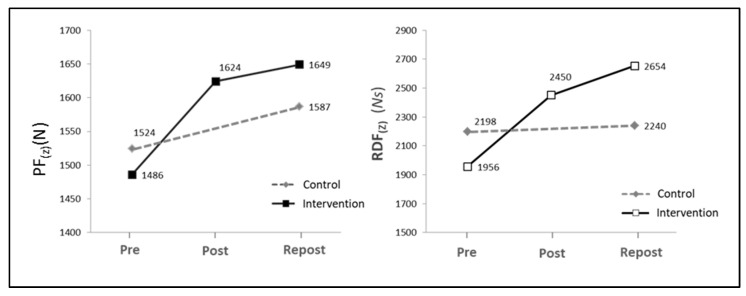
Graphical representation of mean values obtained in the three assessment periods (Pre, Post and Repost) for vertical peak force (PF_(Z)_) and their associated rate of force development (RFD_(Z)_).

Figure 1 shows measures of central tendency for the static force test. Clear differences were observed in vertical peak force (PF_(Z)_) among the means of the Pre-, Post- and Repost measurements made in the intervention group (*F* = 13.97; *p* < 0.001). The hypothesis test revealed that the mean (PF_(Z)_) values increased significantly in the Post- and Repost assessments performed after the intervention (9.3% and 11% for Post and Repost, respectively), as compared to the baseline assessment (Pre). In contrast, no significant differences were obtained in the mean values for Post and Repost. In the control group, the Post and Repost mean values tended to increase with respect to baseline values, without statistically significant differences between mean values for Pre and Repost. The rate of force development (RFD_(Z)_) showed a tendency similar to that of PF_(Z)_, with a lower level of significance (*F* = 5.06; *p* < 0.05). No statistically significant differences were observed in the mean values for Pre and Repost in the control group. 

Table 2 shows measures of central tendency for normal and Dual Task Cost (DTC%) gait in the intervention group. No statistically significant differences were observed in mean values for Pre, Post or Repost (gait cycle time). The mean double-support time (double-support time) tended to decrease significantly after the intervention (7.1% and 8.9% for Post and Repost, respectively) as compared to Pre (*p* < 0.05). Conversely, the mean values for single-support time (single-support time) tended to increase after the intervention (*p* < 0.01). Furthermore, differences only reached significance after 24 weeks of intervention (2.1% in Repost), as compared to baseline values (Pre) in single-support time. The mean dual-task cost for all the time records (DTC%) remained negative. This indicates that the mean time values were higher in the dual-task gait as compared to the normal gait. No evidence was obtained that the intervention program had any effect on the DTC for gait cycle time.

In the intervention group, mean step length (step length) tended to increase after the intervention (*p* < 0.01), although differences only reached significance after 24 weeks of intervention (4.4% in Repost), as compared to baseline values (Pre). The dual-task cost (DTC%) remained positive in the three assessment periods, which indicates that the step length was lower in the dual-task gait as compared to normal gait. The mean DTC% value decreased significantly after the intervention (Post and Repost), as compared to mean baseline value (Pre) (*p* < 0.05). The mean CM velocity along the gait cycle (*Cycle_CM_V*) increased after the intervention, although differences were only significant for mean CM velocity in the double-support phase (double support *_CM_ V*) (5.4% of Pre value; *p* < 0.05). The mean DTC values (DTC%) for mean CM velocity decreased after the intervention (*p* < 0.05), although differences only reached significance after 24 weeks of intervention, as compared to baseline values.

**Figure 2:**
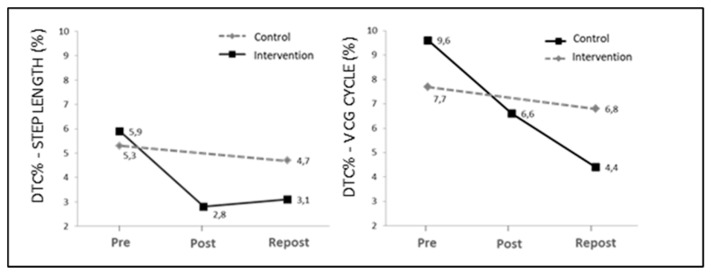
Graph of the mean values for the three assessments (Pre, Post and Repost) for the DTC for step length and gait cycle velocity (DTC%-_STEP LENGTH_ and DTC%-V_CM CYCLE_, respectively).

In the control group, no statistically significant differences were observed between the mean values of gait parameters. The same is seen for the DTC of the concurrent cognitive task. Figure 2 shows DTC values for step length and mean gait velocity (DTC%_STEP LENGTH_ and DTC%-V_CM CYCLE_, respectively) for the intervention and control group. 

Table 3 displays the measures of central tendency for stabilometric parameters in the intervention group. In general, all mean values decreased after the intervention (Post and Repost) as compared to pre-intervention values (Pre). Furthermore, not all differences were significant. Differences between mean values were only significant in anteroposterior displacement (AP_(PC)_; *p* < 0.001) and total stride length (L_(CP)TOTAL_; *p* < 0.01) for the condition with the highest instability, i.e., on foam with eyes closed (RFCE). In the control group, no statistically significant differences were observed among the mean values of the stabilometric parameters in the four experimental conditions.

Table 4 contains measures of central tendency for the sit-to-stand (STS) parameters in the intervention group. The mean peak values for the vertical component of reaction forces (PF_(Z)_) increased after the intervention (6.1% and 10% for Post and Repost, as compared to Pre, respectively; *p* < 0.01), although the differences in mean values between Post and Repost were not statistically significant. Similar results were obtained for the horizontal component (PF_(X)_). Mean peak reaction force values for the two components also increased in Repost as compared to Pre, although they did not reach statistical significance. 

**Figure 3:**
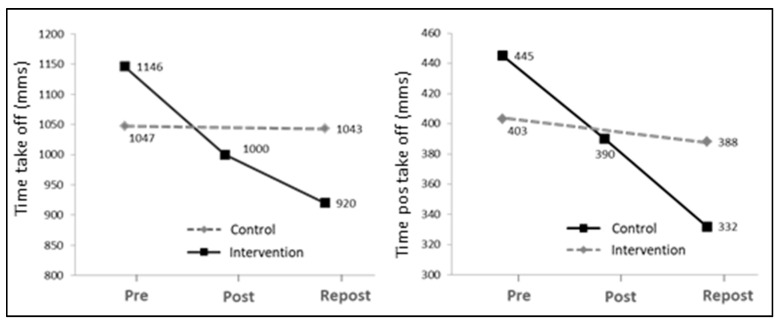
Graph displaying mean values in the three assessment periods (Pre, Post and Repost) for time to complete STS (stand-up t.) and the time interval until take-off (take-off t.).

The mean STS time decreased significantly after the intervention (12.7% and 19.7% for Post and Repost, respectively), as compared to baseline values (Pre) (*p* < 0.01). Values primarily decreased in the interval between onset and take-off (Time take-off; *p* < 0.001) and increased after 24 weeks of intervention (Repost) as compared to 12 weeks (Post). As shown in Figure 3, no statistically significant differences were found in the mean time values among the three assessment periods.

The mean minimal vertical velocity of the CM (*v*_CM(Z) MIN_) decreased after the intervention (*p* < 0.05). However, the differences were only significant in the mean values between baseline assessment (Pre) and at 24 weeks (30% in Repost). The vertical velocity of the CM at take-off (*v*_CM (Z)_ take-off) increased after the intervention (Post and Repos) as compared to baseline values (Pre) (*p* < 0.001). In the control group, no statistically significant differences were observed in mean values between Pre and Repost.

## 4. Discussion

The aim of this study was to assess the effects of a combined training program (CTP), combining strength exercises and cognitive–motor tasks performed concurrently in participants with multiple sclerosis, and compare the results with a matched control group that received standard rehabilitation. The global results suggest that engaging in a CTP program for 24 weeks helps MS subjects improve muscle strength, the rate of force development and balance, as compared to controls. These results are not striking, as the 24-week CTP program was aimed at improving these skills. The results of our study are in agreement with those of previous studies based on similar intervention programs [5,6,13,22,23]. This study demonstrates the benefits that CTPs have for daily activities, such as walking or sitting-to-standing. Furthermore, this study shows that CTPs reduce the dual-task cost of performing cognitive tasks while walking (DTC%). 

Although CTP did not cause significant differences in all conditions and stabilometric parameters, the data obtained show a generalized improvement of balance; specifically, an enhanced somatosensory response (RFCE-RFOE). This parameter indicates to what extent vision is used in postural control when somatosensory afferences are restricted. This general improvement in balance might be associated with the incorporation of a battery of exercises on instable plates in the CTP. Nevertheless, the improvement of postural control under instability conditions might also be due to an enhanced rate of force development (RFD), which is associated with a higher rate of muscle strength production. This could have contributed to postural control under conditions of instability, where somatosensory afferences were restricted. This could be consistent with the contributions of Fimland et al. [23], who documented an increase in the neural drive of MS subjects following a strength training program. 

The data obtained show scarce differences in mean stride time values among the experimental conditions. Yet significant differences were observed in the time parameters in the phases of gait, with a reduction in double-support time values (*p* < 0.05) and an increase in single-support time values (*p* < 0.01). The lower time values recorded in the double-support phase could be related to an improvement in dynamic balance and postural control, as proposed by Kalron et al. [26] and Davies et al. [27]. This is supported by the data obtained via the Romberg test (see Table 3). As a result, the mean velocity of the CM increased during this phase. The increased time values in the single-support phase after the intervention could be associated with the significant increase in mean step length (see Table 2).

There is consistent evidence proving the effects that concurrent cognitive tasks (dual tasking) have on gait performance. In dual tasking, tasks compete for resources and processes, which causes significant changes in the gait pattern of MS subjects with neurological impairment [14,21]. The data exposed in this study confirm this hypothesis, as step length decreased by a mean of 5.5% as a result of dual tasking, as compared to normal walking (DTC% = 5.9% and 5.3% for Pre values in the intervention group and controls, respectively). However, after CTP, the intervention group reduced this difference by up to 3.1%, while controls maintained baseline values (4.7%). 

Similar results were obtained for the mean velocity of the CM along the gait cycle and its phases (see Figure 2). The reductions in DTC% may be related to improvements in static force and balance, which is consistent with the results of Yahia et al. [28] and Broekmans et al. [29]. These authors suggested that deficit of strength in MS subjects could have negative effects on daily activities such as walking, climbing stairs or sitting and standing. However, further studies are needed to separately determine the specific effect of the dual-task training included in our CTP on neural plasticity, and on the cognitive and motor processes that affect motor inhibition and reprogramming for adaptation to a new situation in dual-task walking.

The most significant improvements elicited by our CTP were achieved in sit-to-stand tests. Our results suggest that the main improvement factor is associated with the increase of general dynamic strength and, to a lesser extent, coordination factors related to postural control. Thus, the 10% increase in reaction dynamic force components after the CTP may have increased CM velocity from onset to take-off. As a result, the vertical velocity of the CM at take-off increased (*p* < 0.001). At the onset of the sit-to-stand motion, the vertical velocity of the CM reached negative values in all assessment periods (_vCM (Z) MIN_). This suggests that subjects flexed the trunk prior to take-off to facilitate the subsequent knee and hip extension. Our data show that negative values in *v*_CM (Z) MIN_ tended to decrease with the CTP. This may be explained by the reduced initial trunk flexion achieved with the CTP. Trunk flexion may have been reduced by the higher initial momentum caused by the increased strength in lower-limb extensor muscles. As a result, joint coordination would improve in sit-to-stand exercises, as proposed by Fujimoto and Chou [30] and Bowser et al. [31]. Therefore, the CTP helped MS subjects improve their performance in sit-to-stand tests as a result of the improvement achieved in dynamic strength. 

## 5. Conclusions

The results obtained demonstrate that MS subjects who engage in a 24-week CTP improve their balance, rate of force development and static strength muscles, as compared to MS controls. CTP could improve MS subjects’ performance in daily activities, such as walking or sitting and standing.

The most significant improvement was observed in the Romberg coefficient, with reduced somatosensory impairment (RFCE-RFOE). Apart from the effects of training on an instable surface, postural control may have improved as a result of the enhanced neural drive associated with the increased rate of force development achieved with the CTP. 

After the 24-week CTP, the dual-task cost to gait performance decreased by 54% (CM velocity) in the intervention group, as compared to controls. Further studies with large sample sizes are necessary to compare different exercise alternatives, so as to confirm the separate effects of each of the dual-task training exercises included in our CTP on the cognitive and motor processes related to motor inhibition and reprogramming that dual tasking requires. 

The relevance of this research is that it has made it possible to verify the effects of a training program that has been designed to improve the specific deficits that MS entails in its different degrees of disability, using complex quantitative biomechanical techniques that have great reliability, validity and precision in measurement. This research shows that patients with MS might benefit from the addition of training programs to their rehabilitation plan.

## Figures and Tables

**Table 1:** Characteristics of the subjects in each group.

	Intervention Group *(N = 15)*	Control Group *(N = 11)*	*p* value
Age (years)	40.7 ± 8.2 (55–26)	47.2 ± 9.8 (61–31)	*n.s.*
Height (m)	1.70 ± 0.09 (1.84–1.55)	1.68 ± 0.07 (1.76–1.55)	*n.s*
Weight (Kg)	72.4 ± 14.7 (107–50)	68 ± 15.1 (100–51)	*n.s*
Skeletal muscle mass (Kg)	26.7 ± 6.4 (37.6–17.5)	26.0 ± 6.9 (40.9–18.8)	*n.s*
Skeletal muscle mass (%)	37.3 ± 7.1 (48.2–34.1)	38.7 ± 7.5 (48.1–21.5)	*n.s*
Body fat mass (Kg)	21.0 ± 7.0 (33.8–9.2)	20.2 ± 7.2 (27.0–10.2)	*n.s*
Body fat mass (%)	29.1 ± 7.2 (35.9–13.9)	20.0 ± 6.8 (35.9–18.6)	*n.s*
Body mass index (Kg/m^2^)	24.7 ± 3.8 (34.5–20.4)	24.9 ± 4.8 (34.5–19.0)	*n.s*
Quality of life (%)	72 ± 6 (80 ± 65)	73 ± 7(84 ± 64)	*n.s*
EDSS	3.6 ± 1.8 (6–1)	3.8 ± 1.2 (6–2)	*n.s*

Mean ± SD (Range); *n.s.* = *p* > 0.05.

**Table 2:** Descriptive and inferential statistics for gait cycle time parameters in the three evaluations in the intervention group.

Variables	Pre	Post	Re-Post	F
Gait Cycle Time, GCT (s) (ms)	1085 (1230 to 880)	1063 (1221 to 872)	1059 (1169 to 900)	1.92
DTC% (%)	−4.8 (1.7 to −18.7)	−4.53 (0.9 to −15.6)	−1.72 (4.3 to −4.6)	2.23
double-support time (ms)	169 (235 to122)	157 (230 to 115) ^1^	154 (200 to 115) ^1^	5.39 *
DTC% (%)	−11.7 (0 to −32.6)	−12.7 (0 to −34.1)	−12.1 (7.8 to −21.7)	0.1
single-support time (ms)	373 (415 to 320)	375 (435 to 320)	381 (420 to 330) ^1,2^	6.61 **
DTC% (%)	−0.4 (7.2 to −5.8)	−0.3 (8.3 to −10.1)	−0.4 (9.9 to −11.9)	0.08
Step length (m)	0.68 (0.89 to 0.38)	0.70 (0.882 to 0.46)	0.71 (0.91 to 0.5) ^1^	4.59 *
DTC Step Length % (%)	5.9 (15.7 to 0)	2.8 (9.6 to −3.1) ^1^	3.1 (7.4 to −2.1) ^1^	3.69 *
***V***_CM_ cycle (ms^−1^)	1.43 (2.03 to 0.74)	1.50 (2.01 to 0.87) ^1^	1.49 (1.96 to 0.87)	2.9
DTC%-VCM CYCLE (%)	9.6 (28.7 to −0.1)	6.6 (17.7 to −3.7)	4.4 (12.1 to −2.1) ^1^	3.72 *
***V***_CG_ double-support (ms^−1^)	1.49 (2.14 to 0.82)	1.58 (2.15 to 0.92) ^1^	1.57 (2.11 to 0.87) ^1^	4.48 *
DTC% (%)	10.5 (33.9 to −0.8)	6.7 (16.1 to −3.1)	5.0 (14.4 to −6.1) ^1^	3.36 *
***V***_CG_ single-support (ms^−1^)	1.36 (1.93 to 0.66)	1.42 (1.86 to 0.77)	1.41 (1.78 to 0.80)	1.61
DTC% (%)	8.7 (23.4 to −1.8)	6.4 (19.2 to −4.3)	3.8 (10.0 to −2.1) ^1^	3.44 *

Mean (range); ** *p* < 0.01; * *p* < 0.05. ^1^, statistical significant difference with respect Pre; ^2^, statistical significant difference with respect Post.

**Table 3:** Descriptive and inferential statistics for stabilometric parameters in the three evaluations in the intervention group (ROE = Romberg Open Eyes; RCE = Romberg Closed Eyes; RFOE = Romberg Foam Open Eyes; RFCE = Romberg Foam Closed Eyes).

Variables		Pre	Post	Re-Post	F
**ROE** **(*mm*)**	AP_(CP)_	40 (133 to 15)	34.8 (121 to 9) ^1^	38.0 (112 to 17)	4.64 *
	ML_(CP)_	29 (124 to 8)	26 (111 to 8)	25 (100 to 4)	2.20
	L_(CP)TOTAL_	610 (1600 to300)	524 (1200 to 200) ^1^	557 (1200 to 280)	4.05 *
**RCE** (*mm*)	AP_(CP)_	60 (167 to 23)	58 (160 to 14)	53 (127 to 23)	0.62
	ML_(CP)_	53 (145 to 16)	46 (136 to 10)	43 (125 to 12) ^1^	3.49 *
	L_(CP)TOTAL_	971 (2200 to 340)	853 (1900 to 200)	879 (2080 to 380)	1.5
**RFOE** (*mm*)	AP_(CP)_	52 (104 to 9)	50 (99 to 32)	49 (101 to 25)	0.65
	ML_(CP)_	5.9 (102 to 8)	2.8 (96 to 20)	3.8 (68 to 16)	0.05
	L_(CP)TOTAL_	940 (2000 to 380)	824 (2200 to 340)	811 (2040 to 360)	2.23
**RFCE** (*mm*)	AP_(CP)_	109 (204 to 67)	87 (199 to 56) ^1^	85 (164 to 54) ^1^	17.31 ***
	ML_(CP)_	97 (190 to 34)	78 (150 to 36) ^1^	77 (152 to 23) ^1^	4.12 *
	L_(CP)TOTAL_	2352 (4560 to 1200)	1674 (3400 to 800) ^1^	1827 (3800 to 740) ^1^	9.1 **

Mean (range); *** *p* < 0.001; ** *p* < 0.01; * *p*< 0.05. ^1^, statistical significant difference with respect Pre; ^2^, statistical significant difference with respect Pos.

**Table 4:** Descriptive and inferential statistics for Sit-to-Stand (STS) task parameters in the three evaluations in the intervention group.

Variables	Pre	Post	Re-Post	F
Peak Force_(Z),_ PF_(z)_, (N)	936.5 (1394 to 702)	994.7 (1485 to 797) ^1^	1029.9 (1469 to 715)^1^	8.03 **
Peak Force _N(Z)_ (N)	222.8 (344 to 113)	280.9 (435 to 97) ^1^	316.1 (464 to 130) ^1^	8.03 **
Peak Force _(X),_ PF_(x)_, (N)	113.8 (193 to 45)	159.9 (223 to 64) ^1^	143.2 (219 to 67) ^1^	5.99 **
STS task time (*mms*)	1146 (1898 to 852)	1000 (1880 to 721) ^1^	920 (1488 to 706) ^1^	6.17 **
Time take-off (*mms*)	445 (776 to 280)	390 (596 to 276) ^1^	332 (414 to 244) ^1,2^	9.56 ***
Time pos-take-off (*mms*)	702 (776 to 280)	611 (596 to 276)	588 (414 to 244) ^1^	3.16
*v*_CM (Z) MAX._ (*ms^−1^*)	0.79 (1.01 to 0.47)	0.81 (1.01 to 0.53)	0.83 (1.03 to 0.49)	0.41
*v*_CM (Z) MIN._ (*ms^−1^*)	–0.05 (0 to –0.13)	–0.04 (0 to –0.1)	–0.03 (0 to –0.07) ^1^	5.44 *
*v*_CM (Z)_ take-off (*ms^−1^*)	0.14 (0.27 to 0.02)	0.22 (0.55 to 0.01) ^1^	0.27 (0.43 to 0.09) ^1^	9.82 ***
*v*_CM (X)_ take-off (*ms^−1^*)	0.37 (0.55 to 0.13)	0.41 (0.53 to 0.16) ^1^	0.38 (0.60 to 0.19)	1.45

Mean (range); *** *p* < 0.001; ** *p* < 0.01; * *p* < 0.05. ^1^, statistical significant difference with respect Pre; ^2^, statistical significant difference with respect Pos.
